# Fluorescence diffuse optical monitoring of bioreactors: a hybrid deep learning and model-based approach for tomography

**DOI:** 10.1364/BOE.529884

**Published:** 2024-08-02

**Authors:** Jiaming Cao, Jon Gorecki, Robin Dale, Chileab Redwood-Sawyerr, Cleo Kontoravdi, Karen Polizzi, Christopher J. Rowlands, Hamid Dehghani

**Affiliations:** 1 School of Computer Science, University of Birmingham, Birmingham B15 2TT, United Kingdom; 2 Department of Bioengineering, Imperial College London, London SW7 2AZ, United Kingdom; 3 Department of Chemical Engineering, Imperial College London, London SW7 2AZ, United Kingdom

## Abstract

Biosynthesis in bioreactors plays a vital role in many applications, but tools for accurate *in situ* monitoring of the cells are still lacking. By engineering the cells such that their conditions are reported through fluorescence, it is possible to fill in the gap using fluorescence diffuse optical tomography (fDOT). However, the spatial accuracy of the reconstruction can still be limited, due to e.g. undersampling and inaccurate estimation of the optical properties. Utilizing controlled phantom studies, we use a two-step hybrid approach, where a preliminary fDOT result is first obtained using the classic model-based optimization, and then enhanced using a neural network. We show in this paper using both simulated and phantom experiments that the proposed method can lead to a 8-fold improvement (Intersection over Union) of fluorescence inclusion reconstruction in noisy conditions, at the same speed of conventional neural network-based methods. This is an important step towards our ultimate goal of fDOT monitoring of bioreactors.

## 1. Introduction

Biosynthesis through the fermentation of cell cultures in bioreactors has made major contributions to e.g. biopharmaceutical and waste treatment processes, and has been playing an important role in the economy [1]. Conventionally, monitoring cell health in bioreactors is done through off-line and at-line methods, both of which involve manual sampling of the cell culture [2]. This is not only operationally tedious but also introduces a delay in the assessment. *In situ* monitoring, or in-line monitoring as referred to by the bioreactor community, has emerged in recent years to enable efficient real-time assessment of cell growth. Techniques such as near-infrared [3], mid-infrared [4], and Raman spectroscopy [5] have shown great success, but all of these methods have a crucial limitation: they fail to resolve the spatial distribution of the cells’ properties, including concentration.

Utilizing a bioreactor, it is possible to monitor cells’ local conditions as reported through the fluorescence strength and hence monitor their distribution using fluorescence diffuse optical tomography (fDOT). In brief, the bioreactor is illuminated at the “excitation wavelength” $\left(\lambda_{x}\right)$ , and the activated cells consequently fluoresce at a different “emission wavelength” $\left(\lambda_{m}\right)$ . Boundary measurements are taken at both $\lambda_{x}$ and $\lambda_{m}$ , and together they can be used to tomographically reconstruct the fluorescence strength and distribution in the bioreactor, and therefore the conditions of the cells. The principles of fDOT are detailed in Section 2.1.

We aim to accurately reconstruct the macroscopic spatial distribution of the fluorophores using fDOT, but this is faced with several challenges. Firstly, though excellent reconstruction results have been reported [6–8], a dense grid of measurements is typically required. This is however impractical in our proposed system: the bioreactor is 13 cm in diameter and 15 cm in height, and while it is possible to sample the whole space with high density, processing the data quickly becomes computationally intractable. Like its DOT cousin, insufficient spatial sampling can give rise to undesirable artifacts [9], which limits the accuracy of the reconstruction. In addition, it is difficult to accurately estimate the background optical properties of the cell culture in the bioreactor, and a mismatch of optical properties between the system and the algorithm is another known source of fDOT artifacts [10]. It is hence important to develop a method that robustly results in accurate fDOT reconstructions under non-ideal conditions.

The use of neural networks in tomographic reconstructions is already well-supported in the literature [11–13]. Despite the varying internal structures, a classic architecture largely contains two parts: first a fully connected layer that projects the sensor-space data to source space, and then a network that refines the projection into the final reconstruction. As is derived in [14], this is equivalent to first performing a “coarse” reconstruction (the fully connected layer) and then denoising it (the rest of the network). Analogous to the 2D application in photoacoustic tomography [15], in this work, we draw inspiration from this proof and propose a two-step hybrid approach, where we first use the classic normalized-Born ratio method to obtain a preliminary reconstruction result that may contain artifacts, and subsequently refine it using a trained network. While this is mathematically equivalent to the conventional neural network-based methods, which promises the same accurate reconstructions, we have the advantage of having extra control over the intermediate step and importantly, considerably saving training time. Less training is required because, without the fully connected layer, the number of weights to train is reduced by *millions*: if we make a conservative estimation and suppose that a fDOT system has 100 channels and is discretized into 10,000 nodes, the fully connected layer should then contain $10^{6}$ connections.

In comparison to fDOT imaging in biological tissues where hand-tuned model-based approaches are typically used [16,17], one unique challenge of bioreactor monitoring is the need to accurately estimate the distribution of the cells, which can benefit from data-driven approaches. This further motivates our two-step hybrid approach, where a data-driven (i.e. a neural network in this paper) approach is used on top of the classical algorithms to enhance the results.

In Section 2, we first briefly overview the principles of fDOT and the classic normalized-Born ratio method, and then present the architecture of the neural network that we used. The proposed method was first validated using simulated data and then a phantom experiment where the locations of the fluorophores were known. The experiment designs are detailed in the second half of Section 2, and the results are presented in Section 3. Finally, the potential caveats and future work are discussed in detail in Section 4.

## 2. Methods

### 2.1 Theory

The fDOT problem is governed by a set of coupled diffusion equations [18], which in the continuous wave case states,

(1) $$-\nabla \cdot \kappa_{x}(r)\nabla \Phi_{x}(r)+\mu_{ax}(r)\Phi_{x}(r)=q_{0}(r)$$

(2) $$-\nabla \cdot \kappa_{m}(r)\nabla \Phi_{m}(r)+\mu_{am}(r)\Phi_{m}(r)=\Phi_{x}(r)\eta \mu_{af}(r)$$

where subscripts *x* and *m* indicate the properties at $\lambda_{x}$ and $\lambda_{m}$ , respectively. Among the intrinsic parameters, $\mu_{a*}(*=x\text{ or }m)$ are the absorption coefficients at the excitation and emission wavelengths, r is the location vector, $\Phi_{*}(r)$ is the fluence rate, $q_{0}(r)$ is the isotropic source, and $\kappa_{*}(r)=\frac{1}{3(\mu_{a*}+\mu_{s*}^{\prime })}$ is the diffusion coefficient where $\mu_{s*}^{\prime }$ denotes the reduced scattering coefficient. Among the fluorescence parameters, η is the quantum efficiency, and $\mu_{af}(r)$ is the absorption coefficient of the fluorophore. Note that it usually suffices to treat $\eta \mu_{af}(r)$ as a lumped term. We hereafter denote it as $\gamma (r)=\eta \mu_{af}(r)$ for convenience, which is also known as the quantum yield. The boundary condition is typically chosen to the Robin type [16,18], which states, for both Eq. 1 and Eq. 2 (* is *x* or *m*, respectively),

(3) $$\Phi_{*}(\xi )+2A\kappa_{*}(\xi )\frac{\partial \Phi_{*}(\xi )}{\partial \hat{\nu }}=0$$

where ξ is a point on the boundary, *A* is a constant related to the refractive indices of the medium and the environment, and $\hat{\nu }$ is the outer normal of the boundary at ξ [19].

In typical fDOT problems, the intrinsic optical properties are assumed to be known, e.g. through prior knowledge or quantification using time-resolved measurements before solving the tomography problem. The goal of fDOT is then to reconstruct γ(r) using the boundary measurements of $\Phi_{x}$ and $\Phi_{m}$ . It is usually necessary to obtain multiple sets of measurements by changing $q_{0}$ for a good reconstruction result.

### 2.2 Classic normalized-Born ratio method

For a pair of excitation source and fluorescence detector located at $r_{s}$ and $r_{d}$ , the Born formulation states [16],

(4) $${\hat{\Phi }}_{m}(r_{s},r_{d})=\int d^{3}r\;{\bar{\Phi }}_{x}(r_{s},r)G_{m}(r_{d},r)\gamma (r)$$

where ${\hat{\Phi }}_{m/x}(r_{s},r_{d})$ and ${\hat{\Phi }}_{m/x}(r_{s},r_{d})$ denote the *measured* fluence rate at location r inside the medium and at the detector, respectively, given an excitation source at $r_{s}$ , ${\bar{\Phi }}_{m/x}(r_{s},r)$ and ${\bar{\Phi }}_{m/x}(r_{s},r_{d})$ denote the same but *modelled*, and $G_{m}(r_{d},r)$ is the Green’s function appropriate to Eq. 2.

To eliminate the effects of systematic inhomogeneities e.g. coupling loss, detector gain, and in this paper, loss due to the mirrors (see Section 2.5.1), one classical solution is to normalize each fluorescence recording to the excitation recording of the same source-detector pair [16,20]. This is based on the assumption that these systematic inhomogeneities can be modeled by a multiplication factor that is approximately the same at both $\lambda_{x}$ and $\lambda_{m}$ , i.e. ${\hat{\Phi }}_{m}(r_{s},r_{d})=A_{m}(r_{s},r_{d}){\bar{\Phi }}_{m}(r_{s},r_{d})$ , ${\hat{\Phi }}_{x}(r_{s},r_{d})=A_{x}(r_{s},r_{d}){\bar{\Phi }}_{x}(r_{s},r_{d})$ , and $A_{m}(r_{s},r_{d})\approx A_{x}(r_{s},r_{d})$ . This gives,

(5) $$\begin{array}{ll} \frac{{\hat{\Phi }}_{m}(r_{s},r_{d})}{{\hat{\Phi }}_{x}(r_{s},r_{d})} & \approx \frac{{\bar{\Phi }}_{m}(r_{s},r_{d})}{{\bar{\Phi }}_{x}(r_{s},r_{d})}\\ & =\int d^{3}r\;\frac{{\bar{\Phi }}_{x}(r_{s},r)G_{m}(r_{d},r)}{{\bar{\Phi }}_{x}(r_{s},r_{d})}\gamma (r) \end{array}$$

In discretized (e.g. using finite element method (FEM)) space, Eq. 5 becomes,

(6) $$\frac{{\hat{\Phi }}_{m}(r_{s},r_{d})}{{\hat{\Phi }}_{x}(r_{s},r_{d})}=\sum_{j=1}^{N}\delta V_{j}\frac{{\bar{\Phi }}_{x}(r_{s},r_{j})G_{m}(r_{d},r_{j})}{{\bar{\Phi }}_{x}(r_{s},r_{d})}\gamma_{j}$$

where subscript *j* denotes the *j*-th node, *N* is the total number of nodes, and δV is the support volume at a node. When multiple measurements are made at source-detector pairs $\left(r_{s1},r_{d1}),(r_{s2},r_{d2}),\ldots ,(r_{sM},r_{dM}\right)$ , the discretized forward problem can be represented in a linear form,

(7) y=Jγ+ϵ

where $y\in R^{M}$ is the normalized measurement vector with $y_{i}=\frac{{\hat{\Phi }}_{m}(r_{si},r_{di})}{{\hat{\Phi }}_{x}(r_{si},r_{di})}$ , $\gamma \in R^{N}$ is the fluorescence property to reconstruct with $\gamma ={\left[\gamma_{1},\ldots ,\gamma_{N}\right]}^{\prime }$ , $\epsilon \in R^{M}$ is the measurement noise, and finally, $J\in R^{M\times N}$ is called the Jacobian matrix with,

(8) $$J_{ij}=\frac{{\bar{\Phi }}_{x}(r_{si},r_{j})G_{m}(r_{di},r_{j})}{{\bar{\Phi }}_{x}(r_{si},r_{d})}\delta V_{j}$$

which can be efficiently computed using FEM solvers such as NIRFAST [19]. Typically, we have M≪N , making the system highly underdetermined. The fDOT problem therefore becomes solving the underdetermined linear system. Due to the use of the Born formulation and the self-normalization step, this procedure is conventionally called the “normalized Born ratio” method.

There exist a variety of methods to solve an underdetermined linear system. Here we adopt the simple yet effective Tikhonov regularization, which solves,

(9) $$\hat{\gamma }=\text{arg}\;\min_{\gamma }{||y-J\gamma ||}_{2}^{2}+\alpha {||\gamma ||}_{2}^{2}$$

where α is a scalar hyperparameter that is to be determined per dataset. Conveniently, the problem has a closed-form solution,

(10) $$\hat{\gamma }={\left(J^{T}J+\alpha I\right)}^{-1}J^{T}y$$

In this paper, we modeled the bioreactor as a cylinder with a diameter of 13 cm and a height of 15 cm, which was discretized into a tetrahedral mesh with 114,054 nodes and 589,451 tetrahedrons. ${\bar{\Phi }}_{x}$ and $G_{m}$ were calculated using the NIRFASTer toolbox [19], and then interpolated onto a regular grid with size 48×48×56 , on which the Jacobian (Eq. 8) was calculated. This grid corresponds to a resolution of 2.70 mm in the x−y (radial) direction and 2.68 mm in the *z* (axial) direction. Given each set of measurements, the first-step reconstruction was performed using Eq. 10, and is represented on the grid as volumetric data.

### 2.3 Enhancing the results using a U-Net

**Fig. 1.**
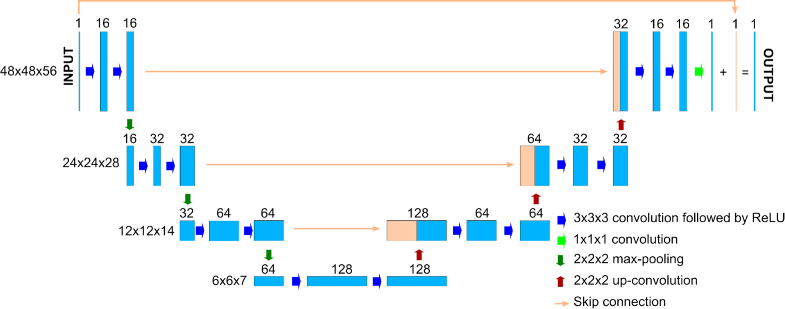
Architecture of the U-Net utilized. The numbers above the blocks indicate the number of channels at each stage, the numbers on the left indicate the size of the 3D images at different layers, and the meanings of the different types of arrows are listed on the bottom right.

The reconstruction result using the classic method was further enhanced using a neural network, which was trained to suppress the artifacts and correct the depth bias. Effectively, this network acts as a denoising filter: its input is the noisy result from the classic algorithm, and its output is the “cleaned-up” version that better estimates the ground truth. Originally developed for segmentation [21], U-Net has also been widely used in image-to-image and volume-to-volume tasks requiring integration of high- and low-level spatial features, including biomedical image denoising [15,22,23]. The architecture of the network used in this paper is similar to that in [15], and is illustrated in Fig. 1.

Analogous to [15], the network utilizes residual learning, which has been shown effective in similar denoising applications [24,25]. A simplistic sum of squares loss was used, and it was computed only on the voxels that are within the cylindrical model. The model was implemented using PyTorch in Python. For both simulation validation and experimental validation, we simulated 2500 instances with varying intrinsic optical properties and inclusions of varying numbers, $\eta \mu_{af}$ , and size. The parameters used in the simulations are detailed in Section 2.4 and Section 2.5.3.

Among the 2500 simulated instances, 100 were reserved as the final testing set and not utilized in the training process. Among the 2400 used in training, 2048 were fed into the optimizer to optimize the network, and the remaining 352 were used as validation data, on which the network was evaluated after each epoch to determine early termination and avoid overfitting. The batch size for training was set to 16, and the maximum number of training epochs was set to 200, with an early termination criterion of validation loss not decreasing for 5 consecutive epochs. All images (both noisy and ground truth) were normalized to their respective standard deviation before being utilized for training the network.

### 2.4 Simulation validation

We first validated our proposed pipeline using simulated data. Specifically, we considered a hypothetical situation where the “bioreactor” has homogeneous, non-fluorescent background optical properties, and the only source of fluorescence was 1 or 2 non-overlapping spherical inclusions. The goal is therefore to reconstruct the sizes and the locations of the fluorescent inclusions. We also hypothesized that the background optical properties $\left(\mu_{ax},\mu_{am},\mu_{sx}^{\prime }\;\text{and}\;\mu_{sm}^{\prime }\right)$ were only *approximately* known, mimicking the real-life situation where these parameters cannot be accurately determined, and that $\mu_{ax}=\mu_{am}=\mu_{a}$ and $\mu_{sx}=\mu_{sm}=\mu_{s}^{\prime }$ . 64 sources $\left(\text{at}\;\lambda_{x}\right)$ and 56 detectors $\left(\text{at}\;\lambda_{m}\right)$ were uniformly placed around the side surface of the “bioreactor”, and measurement pairs were formed wherever a pair of source and detector had a separation of less than 7 cm, forming 2592 channels in total. The model used is illustrated in Fig. 3(c). The parameters used for data generation is summarized in Table 1. The uniform distribution was used for all the randomizations.

**Fig. 2.**
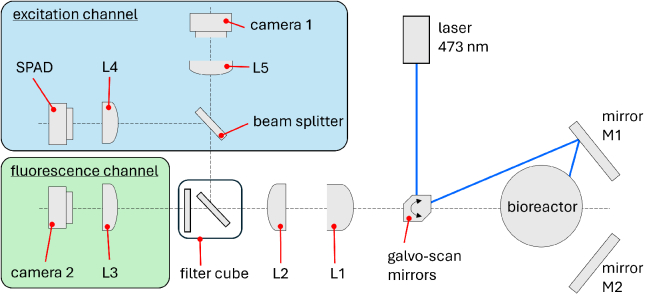
**Schematic of optics setup:** A 473 nm laser is diverted via a pair of scanning-galvanometer mirrors towards the bioreactor vessel. Two mirrors placed behind the vessel allow the beam to be incident on the vessel from all directions. An arrangement of lenses and filters allows for the bioreactor to be imaged in excitation and fluorescence channels.

**Fig. 3.**
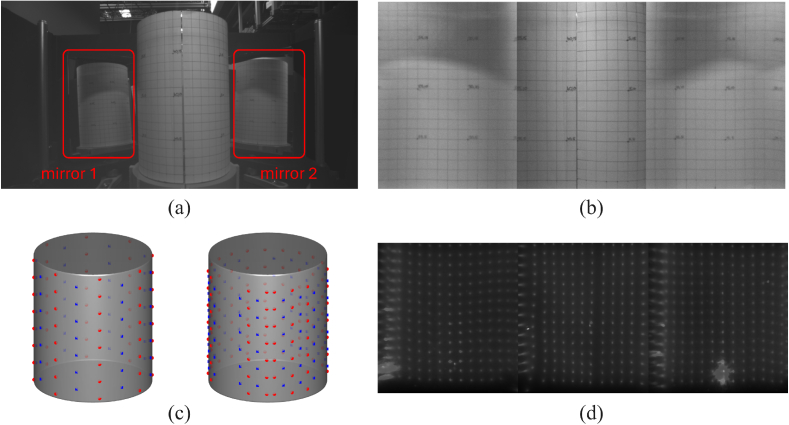
Illustration of mapping camera data to the cylindrical mesh. (a) Raw image from the camera, with the calibration paper grid around the bioreactor (b) Unwrapped image of (a), which was then used to determine the piece-wise linear relationship between pixels and spatial locations (c) The models used for simulation validation (left) and phantom experiment (right). Red: sources; Blue: measurements (d) All unwrapped excitation channel images for Sample A overlaid. The bright spots are the locations of light incidence. The source locations in (c) right are a subset of the bright spots mapped to the cylinder surface. Distortions can also be observed near the boundaries of the three regions, potentially due to the sharp angle of incidence of the illumination beam.

**Table 1.** Parameters used for generating data in the simulation validation.

*μ_a_* (mm^−1^)	0.0089±10%
$\mu_{s}^{\prime }({\text{mm}}^{-1})$	1.314±10%
No. of Spheres	1-2
Radii (mm)	7-15
$\eta \mu_{af}\;({\text{mm}}^{-1})$	$4\times 10^{-4}\;\text{-}\;4\times 10^{-2}$
Noise	0-2%

Note that the background optical properties were chosen to be similar to the default fluorescence meshes provided in NIRFASTer [19], and the noise was added to the Born ratios (i.e. left-hand side of Eq. 6). 0-2% denotes that the absolute values of zero-mean Gaussian noise, whose standard deviation ranged from 0-2% of the maximum amplitude, were used.

The first-step classic reconstruction, i.e. the noisy images fed into the neural network, was then performed using Eq. 10, with α chosen to be 100 and J computed by assuming *μ_ax_*=0.0089 mm^−1^, *μ_am_*=0.0062 mm^−1^, $\mu_{sx}^{\prime }=1.314\;{\text{mm}}^{-1}$ and $\mu_{sm}^{\prime }=1.274\;{\text{mm}}^{-1}$ .

The trained model was evaluated on the 100 reserved testing data. To quantify the improvement, we calculated the intersection-over-union (IoU) metrics for both the input and the output of the network (i.e. classic method and classic method + denoising), which was defined as:

(11) $$\text{IoU}(\text{Recon},\text{Truth})=\frac{|\{\text{Recon}>1\}\cap \{\text{Truth}>0\}|}{|\{\text{Recon}>1\}\cup \{\text{Truth}>0\}|}$$

where |⋅| denotes the cardinality of a set. Unless otherwise stated, all voxels in the cylindrical model were used to calculate IoU . The reconstruction results were thresholded above 1, which is equivalent to thresholding above one standard deviation in the images prior to normalization. This was done to eliminate the impact of the negative values as well as artifacts with small amplitudes. When the reconstruction is perfect, we have IoU(Recon,Truth)=1 , and in general, it is desirable to be as close to 1 as possible.

### 2.5 Experimental validation

#### 2.5.1 System setup

The optics system is comprised of a blue continuous-wave laser emitting at 473 nm with a power range from 1 - 100 mW (Omicron LuxX-HSA), which is incident upon a pair of galvanometer scanning mirrors (galvos) allowing for the beam to be deflected in two axis according to a computer-controlled applied voltage. The laser beam is diverted towards the bioreactor vessel, where it can either address the front surface of the vessel, or reflect off either mirror M1 or M2 to address the rear side of the vessel (see Fig. 2). Light emitted from the bioreactor is collected and focused via a wide field-of-view fixed focal length lens L1 (Edmund 86-569) and passed into scanning tube lens L2 (Thorlabs TTL200MP) which collimated the beam, creating an infinity corrected imaging system. The infinity focused imaging path allows for the optical components such as filters and beam splitters to be placed in the optical path without causing spherical aberrations to the beam. The filter cube splits the light into the excitation and fluorescence channels via the use of a long-pass dichroic (Semrock Di03-R473-t1-25x36) which transmits the fluorescence and reflects the excitation light. The light entering the fluoresence channel is filtered by a further filter (Semrock BLP01-473R-25) to ensure no significant excitation light is transmitted. Light exiting the filter cube in the fluorescence channel is focused via a tube lens L3 (Thorlabs TTL200A) into a monochrome camera (IDS U3-3290SE-M). Light exiting the filter cube in the excitation channel is split via a 50:50 beam-splitter, sending one path of the beam through tube lens L4 (Thorlabs TTL200A) towards the Single Photon Avalanche Diode array (MPD SPC3), abbreviated to SPAD, and the other path through tube lens L5 (Thorlabs TTL200A) into a monochrome camera (IDS U3-3290SE-M). The SPAD measures spatially resolved time gated information, providing the temporal profile of the incident laser pulse at each pixel, which allows for the scattering and absorption coefficients of the material inside bioreactor vessel to be determined. Once the scattering and absorption coefficients are determined, the cameras on the excitation and fluorescence channels are used for the DOT process to determine the spatial distribution of fluorescence intensity within the vessel.

#### 2.5.2 Optical property fitting

In order to successfully reconstruct the fluorophores, the background optical properties must be first estimated with reasonable accuracy. We are particularly interested in the absorption and reduced scattering coefficients, and because $\lambda_{x}$ and $\lambda_{m}$ are similar in our system (473 nm and 530 nm, respectively), we also make the simplifying assumption that $\mu_{ax}=\mu_{am}=\mu_{a}$ and $\mu_{sx}^{\prime }=\mu_{sm}^{\prime }=\mu_{s}^{\prime }$ . The fitting was done using a method similar to [26]. We assume the time-resolved system described above to be linear and time-invariant (LTI). The system response, also known as the instrument response function (IRF) then comprises factors such as laser pulse width, free-space light traveling, and camera responses. The IRF was estimated using the temporal response at the brightest pixel (indicating light incidence) reflected off a reflective object. Specifically, the time trace was smoothed with a 0.5 ns moving window, baseline-shifted to zero, and finally normalized to its highest amplitude, denoted as h(t) .

Time traces from four pixels (1.38, 1.84, 2.30, 2.76 cm away from illumination) in the phantom data were considered, from which two key features were extracted: the peak amplitude $I_{peak}^{\left(i\right)}$ (estimated as the average of the five largest amplitudes) and mean time of flight $t_{mean}^{\left(i\right)}$ , i=1,2,3,4 . The latter is defined as,

(12) $$t_{mean}=\frac{\sum_{j}t_{j}I_{t_{j}}}{\sum_{j}I_{t_{j}}}$$

where $t_{j}$ is the *j*-th time point in the time vector and $I_{t_{j}}$ is the amplitude at time $t_{j}$ .

The fitting of the optical property was performed using a simple grid search. For each pair of $\left(\mu_{a},\mu_{s}^{\prime }\right)$ at each of the source-detector distances $d_{i}$ , the theoretical temporal point spread function (TPSF) was calculated using the analytical solution in semi-infinite media [27]. The same two features, denoted as $I_{peak}^{\prime \left(i\right)}(\mu_{a},\mu_{s}^{\prime })$ and $t_{mean}^{\prime \left(i\right)}(\mu_{a},\mu_{s}^{\prime })$ to differ from their experimental counterparts, were extracted from $h(t)*\text{TPSF}(\mu_{a},\mu_{s}^{\prime },d_{i},t)$ , where ∗ denotes temporal convolution. The grid search then aims to solve the following problem,

(13) $$\begin{array}{ll} {\hat{\mu }}_{a},{\hat{\mu }}_{s}^{\prime }=\text{arg}\;\underset{\mu_{a},\mu_{s}^{\prime }}{\text{min}}\sum_{i=1}^{4}100* & {\left(\left(t_{mean}^{\left(i\right)}-t_{mean}^{\left(1\right)}\right)-\left(t_{mean}^{\prime \left(i\right)}(\mu_{a},\mu_{s}^{\prime })-t_{mean}^{\prime \left(1\right)}(\mu_{a},\mu_{s}^{\prime })\right)\right)}^{2}\\ & +{\left(\log\frac{I_{peak}^{\left(i\right)}}{I_{peak}^{\left(1\right)}}-\log\frac{I_{peak}^{\prime \left(i\right)}(\mu_{a},\mu_{s}^{\prime })}{I_{peak}^{\prime \left(1\right)}(\mu_{a},\mu_{s}^{\prime })}\right)}^{2} \end{array}$$

where the factor 100 was introduced to balance the weight between the two terms. Note that the problem can be further simplified, but we chose to present it in this form to make the meanings of the two terms clear: it is the *relative* flight time and *relative* amplitude with regard to the first location that matters. In our experiments, the grid for $\mu_{a}$ was $10^{-5}-10^{-3}\;{\text{mm}}^{-1}$ with a grid size of $10^{-5}\;{\text{mm}}^{-1}$ , and the grid for $\mu_{s}^{\prime }$ was $0.02-0.2\;{\text{mm}}^{-1}$ , with a grid size of 0.001 mm^−1^. The $\left(\mu_{a},\mu_{s}^{\prime }\right)$ pair that gave the lowest loss in Eq. 13 was deemed the solution to the fitting problem.

#### 2.5.3 Data processing and reconstruction

The raw images from the cameras were first processed into the “unwrapped” format. In brief, the unwrapping algorithm takes as an input the camera image shown in Figure 3(a), isolates the three sections which show the surface of the vessel, transforms the distorted views into a rectilinear form, and joins the three sections together as shown in Figure 3(b).

To create the mapping algorithm, first a 1 cm square grid is printed on paper and attached to the surface of the vessel. The grid is imaged in the camera as shown in Figure 3(a) which shows the front surface of the vessel in the middle, and the rear surfaces of the vessel on each side which can be seen from reflection in the two large mirrors.

For each of these three domains, a separate mapping function is created. To create the mapping function the location of each 1 cm grid intersection in the camera image (in pixel coordinates) is recorded, and a 2D geometric transform function is created in Matlab which maps these pixel values to an idealised rectilinear grid via a polynomial function. The geometric transform is single-valued (each single point in the camera image relates to a single point in the rectilinear grid), and reversible (the transform function can be used to transform from image to rectilinear grid, and from rectilinear grid back to image).

Next, a high-density rectilinear grid is created and the inverse transform is used to convert each point in the rectilinear grid into a location in the camera image. The pixel value at each location of the image is sampled and then plotted back in the rectilinear grid. This creates an unwrapped “flat view” of the bioreactor vessel surface. The process is repeated for all three sections (the front surface and the two rear sections visible in the mirrors) and the three unwrapped images are stitched together as shown in Figure 3(b).

**Fig. 4.**
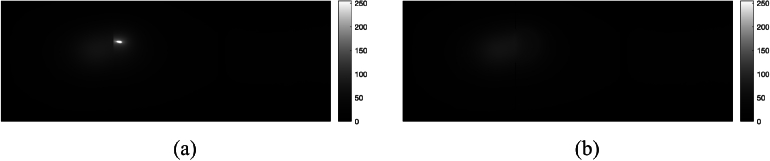
A example of the spot artifact removal. (a) Raw image (b) After spot removal

The small bright spots with saturated pixels in the images (if any) were removed, dilated using a disk with 5-pixel radius, and interpolated. These spots were deemed as artifacts resulting from e.g. fluorescence emission of the vessel itself and spurious reflections, because they were present even when no fluorescence inclusions were inserted. An example is shown in Fig. 4. The resulting images were then further smoothened using a 7×7 median filter.

A cylindrical mesh representing the bioreactor (13 cm diameter and 15 cm height) was created. The sources on the mesh were chosen to be a subset of the illumination locations. Positions where distortion could be observed in our measured data (see Fig. 3(d)) were excluded. Measurement locations were chosen to be a 2 cm-by-2 cm grid wrapped onto the side of the cylinder. Source-measurement separations were constrained to be less than 10 cm. This results in a total of 119 source locations, 102 measurement locations, and 5398 measurement pairs, as is shown on the right-hand side of Fig. 3(c).

Since our data was recorded using cameras, we technically have access to >1,000,000 measurement (i.e. pixel) locations. For computational tractability, we only use a relatively small amount of locations indicated as blue squares in Fig. 3(c). The measurement locations were mapped to the pixel locations in the unwrapped images using the linear relationship described above, and the amplitudes were assigned as the median of the surrounding 9 pixels centered at the mapped pixels. The range of source-measurement separation was intentionally chosen as large, which is assumed to be redundant, and in practice, only a subset of measurements were used depending on the recorded signal strength. In this particular dataset, only channels with ${\hat{\Phi }}_{m}$ greater than 18% of the expected max and ${\hat{\Phi }}_{x}$ greater than 1% of the expected max were used to avoid the impact of quantization noise and camera dark noise.

The classic reconstruction (i.e. noisy images to be enhanced) was then performed using Tikhonov regularization (Eq. 10) with α=1 using only the selected channels, chosen empirically. The same grid described in Section 2.2 was used.

To accommodate for the experimental setup, the neural network was trained again using a new set of data, where 2048 images were used as the training set and another 352 were used as the validation set during training. In the new dataset, the source and measurement grid shown on the right-hand side of Fig. 3(c) was used. Instead of spherical inclusions, cylindrical inclusions with varying radii and heights were used in training data generation. The parameters used are summarized in Table 2.

**Table 2.** Parameters used for generating new data, which were then used to train the neural network again to accommodate the experimental setup.

$\mu_{a}\;({\text{mm}}^{-1})$	0.0005±30%
$\mu_{s}^{\prime }({\text{mm}}^{-1})$	0.1±30%
No. of inclusions	1-3
Radii (mm)	5-10
Heights (mm)	10-30
$\eta \mu_{af}\;({\text{mm}}^{-1})$	$4\times 10^{-4}\text{-}\;4\times 10^{-2}$
Noise at $\lambda_{x}$	0-1%
Noise at $\lambda_{m}$	0-1%

Note that in this dataset, the noise was added individually to the two wavelengths to better represent the experimental measurements. The values used for $\mu_{a}$ and $\mu_{s}^{\prime }$ were fitted using the time-resolved recording (see Section 3.2). When performing the classic reconstruction, only channels with both ${\hat{\Phi }}_{m}$ and ${\hat{\Phi }}_{x}$ greater than 0.8 times the standard deviation of all channels for the respective wavelengths were used.

## 3. Results

### 3.1 Simulated data

**Fig. 5.**
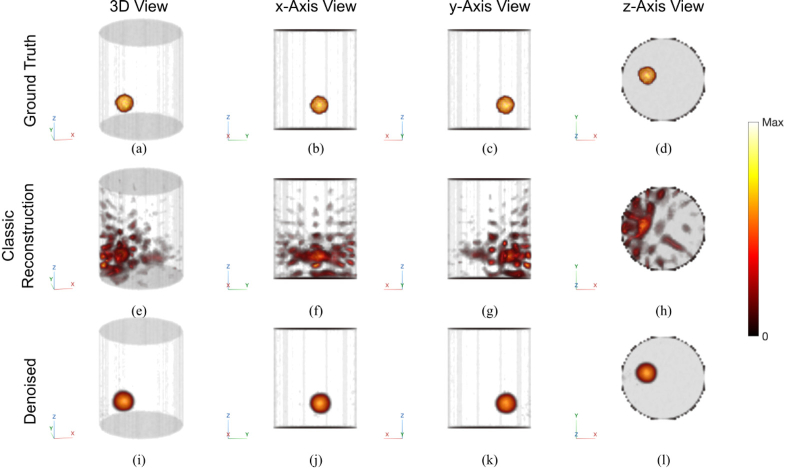
Simulated example of a low noise condition with one inclusion. Although the classic algorithm successfully reconstructed the location of the inclusion, large amounts of artifacts can be seen due to the optical property mismatch and undersampling. These artifacts are almost perfectly eliminated by the denoising U-Net. First row: ground truth; Second row: results of classic algorithm; Third row: results of the denoising U-Net. All reconstruction results are thresholded above 1 (see Section 2.4). Colorbar normalized between minimum and maximum amplitudes.

We first examine an example with one inclusion and low noise. The results are shown in Fig. 5. The optical properties used in forward simulation (Example A) and reconstruction are specified in Table 3. The optical properties were intentionally chosen to be mismatched between data generation and reconstruction, to mimic the inaccurate estimation in experimental situations. The classic reconstruction algorithm ((e)-(h)) performed relatively well despite some artifacts, which are typical due to the mismatched optical properties and sparse sampling [9,10]. The denoising network almost perfectly removed the artifacts, as is shown in Fig. 5(i)-(l). Quantitatively, the IoU metric increased from 0.046 to 0.46.

**Table 3.** Optical properties used in data generation and reconstruction. The same optical properties are assumed for both examples when performing reconstruction.

	$\mu_{ax}\;({\text{mm}}^{-1})$	$\mu_{am}\;({\text{mm}}^{-1})$	$\mu_{sx}^{\prime }\;({\text{mm}}^{-1})$	$\mu_{sm}^{\prime }\;({\text{mm}}^{-1})$	Noise
Example A	0.0090		1.32		0.08%
Example B	0.0085		1.36		1.46%
In Reconstruction	0.0089	0.0062	1.31	1.27	-

**Fig. 6.**
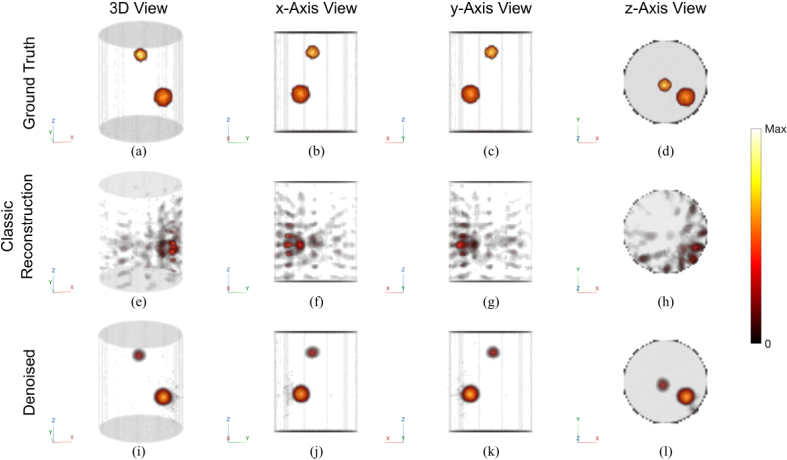
Simulated example of a higher noise condition with two inclusions. The classic algorithm performs worse in this condition and fails to resolve the two inclusions. They are however both successfully recovered by the denoising U-Net. First row: ground truth; Second row: results of classic algorithm; Third row: results of the denoising U-Net. All reconstruction results are thresholded above 1 (see Section 2.4). Colorbar normalized between minimum and maximum amplitudes.

We then examine a more challenging example, where two inclusions and high noise were simulated. The results are shown in Fig. 6. The optical properties used in forward simulation (Example B) and reconstruction are specified in Table 3. The classic approach ((e)-(h)) performed substantially worse, due to both the more drastic mismatch of optical properties and the higher noise. The denoising U-Net ((i)-(l)) successfully recovered both inclusions with little artifact. Quantitatively, the IoU metric increased from 0.053 to 0.66. Although the IoU metric is good, it can be noticed that the size and amplitude of one of the inclusions were slightly underestimated. This can be a result of it being deeper and therefore the sensitivity is lower, highlighting the need for further investigation and potentially more carefully designed training data.

In addition to the two examples shown, we further quantified the improvement using all 100 testing sets using the IoU metric. On average, the metric improved from 0.054±0.026 to 0.44±0.17 , a ∼8 fold improvement. A paired *t*-test produced a *p*-value ≪0.001 , suggesting a statistically significant improvement.

### 3.2 Phantom experiments

A phantom experiment was then conducted to further validate the proposed algorithm. A phantom is created via filament extrusion 3D printing of polylactide (PLA). The phantom consists of a ‘lid’ which sits on the top of the bioreactor vessel which holds 4 vertical rods, each held in a clamp which allows for the height of the rod to be adjusted. Both the lid and rods are printed in a grey non-fluorescent PLA. At the end of each rod is a cylinder which is printed in fluorescent PLA, which is made fluorescent by the inclusion of a dye by the manufacturer. The emission wavelength of the dye was measured to be 530 nm. The rods and cylinders can be set at variable heights within the vessel, or removed completely. The vessel is filled with intralipid and water dilutions which allow for a controllable optical scattering coefficient. One or two 3D-printed, green fluorescent cylinders (2 cm diameter and 3 cm height) were inserted into the liquid phantom as the ground truth. The optical properties of the liquid phantom were fitted to be $\mu_{a}\approx 0.0004\;{\text{mm}}^{-1}$ and $\mu_{s}^{\prime }\approx 0.11\;{\text{mm}}^{-1}$ using the method described in Section 2.5.2 (see Section 4 for further discussion). We nominally chose $\mu_{ax}=\mu_{am}=0.0005\;{\text{mm}}^{-1}$ , $\mu_{sx}^{\prime }=\mu_{sm}^{\prime }=0.1\;{\text{mm}}^{-1}$ for both training data generation and reconstruction.

**Fig. 7.**
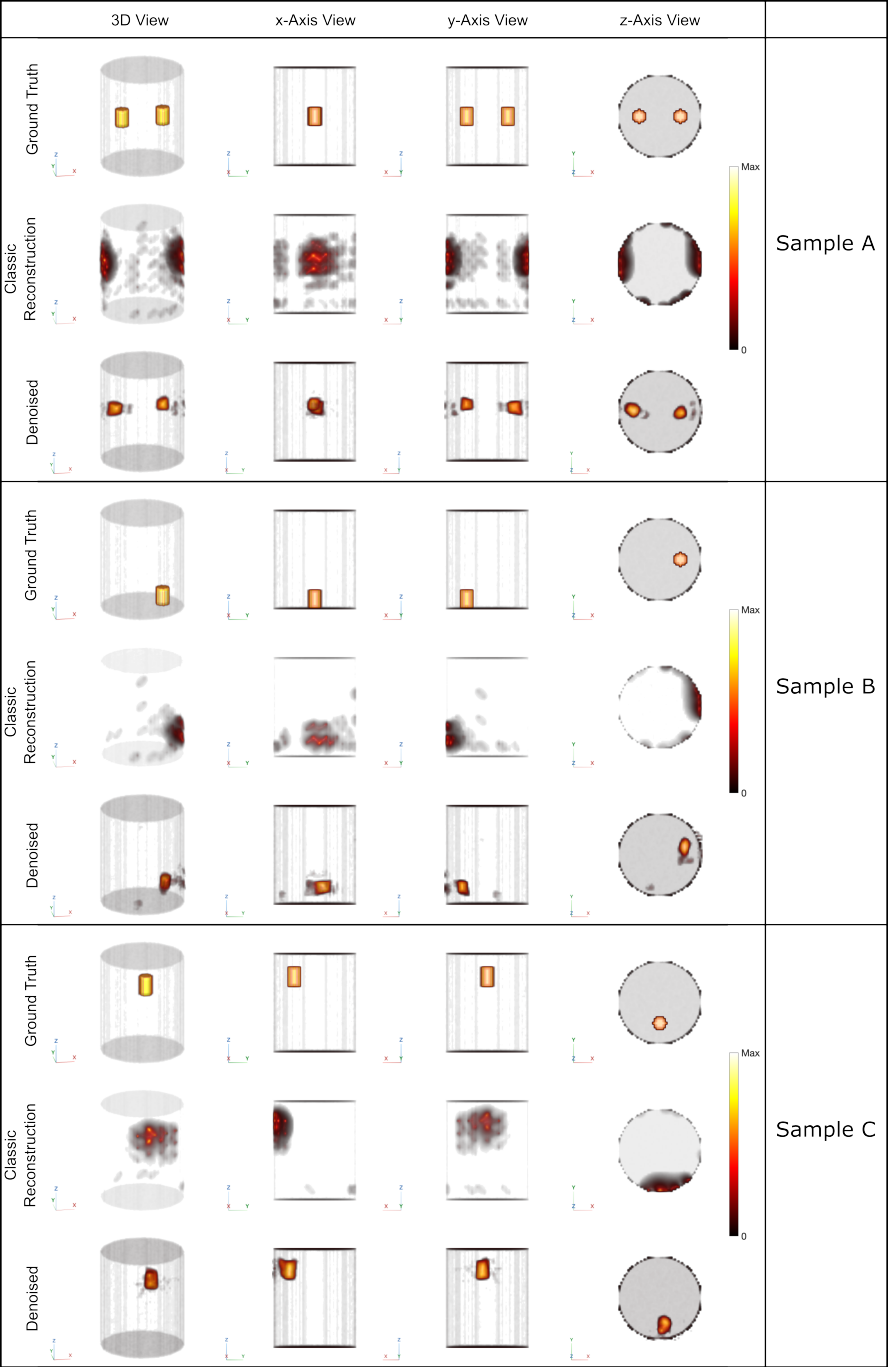
Experimental results: comparison between classic reconstruction and proposed method in three samples. Within each panel, the first row shows the ground truth, the second row shows the results before denoising (i.e. the classic algorithm) and the third row shows the results after denoising using our neural network. While the classic method correctly reconstructs the number of inclusions, the depths of them were incorrect. Furthermore, strong artifacts can be observed. The denoising network successfully eliminates the artifacts and recovers the locations of the inclusions. Colorbar normalized between minimum and maximum amplitudes.

The reconstruction results of three different samples are shown in Fig. 7. The classic method correctly identifies the number of inclusions and their approximate locations, but the results are strongly biased toward the surface and are contaminated with boundary artifacts. The bias exists because when doing the channel selection in the preprocessing step (see Section 2.5.3), only the shorter source-measurement distances remained in our datasets. Our neural network not only drastically reduced the boundary artifacts, but also recovered the depth information which was lost in the classic reconstruction. The comparison shows an excellent agreement between the reconstructed and true locations of the inclusions. The enhanced results were further compared with the ground truth quantitatively, as is shown in Table 4. Four metrics were used: bias of the center of mass, full-width half maximum (FWHM), IoU , and structural similarity (SSIM) index, all of which indicate an improvement in reconstruction accuracy using the neural network. For Sample A, IoU was calculated separately for the two inclusions using the half-cylinder (divided along the y−z plane) containing them, and for Samples B and C, the whole cylinder was used. When calculating SSIM, only the voxels within 3 cm from the true centers of the inclusions were used and the amplitudes were normalized by the maximum. Only subregions were used in order to avoid falsely high indices arising from the background voxels. The IoU values are considerably lower than those in simulated experiments, because of the depth bias and the slightly different geometry of the reconstructed and actual inclusion, which are further exaggerated by the small size of the inclusions.

**Table 4.** Quantitative comparison between the classic and the proposed method using three metrics: bias of the center of mass, full-width half maximum (FWHM), IoU , and SSIM. Bias, IoU and SSIM are calculated using the ground truth illustrated in Fig. 7.

		Sample A		Sample B	Sample C
		Inclusion 1	Inclusion 2		
Bias (mm)	Classic	17.8	21.1	25.2	22.7
	Denoised	10.8	12.1	16.3	12.4
FWHM (mm)	Classic	42.7	33.5	40.9	68.5
	Denoised	18.5	23.0	23.8	33.1
IoU	Classic	0.0066	0.0042	0.0068	0.030
	Denoised	0.17	0.22	0.14	0.17
SSIM	Classic	0.12	0.12	0.25	0.064
	Denoised	0.66	0.64	0.59	0.57

## 4. Discussion and conclusions

In this paper, we proposed a two-step, hybrid method for high-accuracy fDOT reconstruction, where an initial reconstruction is first obtained using the classic algorithm and then enhanced using a denoising U-Net. We demonstrated using both simulated and phantom data that the proposed method can accurately and robustly reconstruct fluorescence inclusions in noisy, sparsely sampled systems. These results support the feasibility of adopting the same technique for our ultimate goal: accurate, *in situ* bioreactor monitoring, a future direction for further work.

To use the proposed method for monitoring actual bioreactors, one challenge is that the cell distribution in a bioreactor is typically a continuum, instead of inclusions in medium, as is modeled in this paper. The training data for the neural network consequently needs to be changed to account for this mismatch. While the proposed method is expected to still perform well e.g. when background fluorescence is present or the distribution of fluorescence is continuous, quantifying the performance of such a scenario remains a subject of future research. We are currently developing approaches that will allow experimental data collection from a more complex known distribution which we hope to report in the near future.

Another challenge is the accurate quantification of the optical properties of the imaged medium. The results of the fitting algorithm (see Section 2.5.2) used in this paper were not validated against known samples, and its accuracy is therefore not quantified. Ideally, the estimation of sample optical properties should be done through a time-domain system [28]. While the accuracy of our fitting may be limited, the impacts of such inaccuracy can be eliminated by incorporating the uncertainty when training the neural network. The fact that good results were still obtained using only approximately determined optical properties demonstrates the robustness of our algorithm.

One limitation of this paper is the number and shape of the simulated inclusions. It remains in future work to systematically quantify the performance of our model in “extrapolated” situations, e.g. when the system has more inclusions than the model is trained for, or when the inclusions are in shapes that are not modeled in the training data. A straightforward solution is to further diversify the numbers and shapes of the inclusions in the training data.

Although the accuracy of the recovered contrast was not quantified within this work, it is expected to perform well as it has been previously demonstrated for DOT [29]. An in-depth investigation of the quantification ability of our method remains a topic of future research.

Although the improvements in reconstruction accuracy are statistically significant, one may notice that the standard deviations of the IoU metrics (in Section 3.1) are fairly large, suggesting that in certain cases, the improvement can in fact be limited. This can be because of the wide range of noise and optical property mismatch introduced, which promotes the generality of the model but at the cost of specificity. With better characterization of the system, it is possible to narrow the range of the fluctuations added to the training data, and a better balance between generality and specificity can be found. This will be investigated in future work.

Among the >5,000 measurement channels modeled when generating training data, only a few hundred were actually used in reconstruction, due to the channel selection. This suggests that at least in situations where inclusions are sparse, spatial sampling can be much more efficient. Strategies such as adaptive sampling, where the sampling density is correlated with the fluorophore density, can potentially further improve the reconstruction results.

One may have the concern that the two-step method is computationally slower than the conventional *sensor data in, reconstruction out* networks. In fact, if the inverse operator ${\left(J^{T}J+\alpha I\right)}^{-1}J^{T}$ in Tikhonov regularization (Eq. 10) is pre-calculated, applying it to data is merely a matrix-vector operation that has O(MN) complexity, where *M* is the number of measurements and *N* is the number of nodes in the mesh. The fully connected layer that projects sensor space data to source space, on the other hand, has MN connections and the complexity is therefore also O(MN) . Assuming the structure of the rest of the neural network is the same, our proposed method should have exactly the same computational complexity as the conventional networks. The two-step pipeline allows one to conveniently incorporate additional knowledge of the system, e.g. spatial priors, into the first step, providing much more flexibility. Of course, manually chosen parameters for the first step reconstruction can also be suboptimal and limit the accuracy of the final result, in which case the conventional networks can be more beneficial. The balance between flexibility and optimality should therefore be carefully considered by the user.

One benefit of our two-step approach is that, as long as the hyperparameters in the model-based reconstruction method are determined, the neural network can be trained again to be optimized for the specific choice of hyperparameters. This suggests that the proposed algorithm is expected to perform well, independent of the hyperparameters.

Using neural networks in hybrid with classic algorithms shows great promise in efficient, accurate, and robust fDOT reconstructions, shedding light on high-accuracy real-time monitoring of bioreactors. To enable this, more careful modeling of the system, e.g. optical properties and cell distribution, is needed. This is to be investigated in our next step.

## Data Availability

The NIRFASTer toolbox used in the work is available open source at [30]. The codes used for data generation, network training, and visualization are available at [31].
